# A comparative study of infrared and microwave heating for microbial decontamination of paprika powder

**DOI:** 10.3389/fmicb.2015.01071

**Published:** 2015-09-30

**Authors:** Lovisa Eliasson, Sven Isaksson, Maria Lövenklev, Lilia Ahrné

**Affiliations:** Food and Bioscience, SP Technical Research Institute of Sweden, Gothenburg, Sweden

**Keywords:** infrared heating, microwave heating, microbial decontamination, paprika powder, quality

## Abstract

There is currently a need in developing new decontamination technologies for spices due to limitations of existing technologies, mainly regarding their effects on spices’ sensory quality. In the search of new decontamination solutions, it is of interest to compare different technologies, to provide the industry with knowledge for taking decisions concerning appropriate decontamination technologies for spices. The present study compares infrared (IR) and microwave decontamination of naturally contaminated paprika powder after adjustment of water activity to 0.88. IR respectively microwave heating was applied to quickly heat up paprika powder to 98°C, after which the paprika sample was transferred to a conventional oven set at 98°C to keep the temperature constant during a holding time up to 20 min. In the present experimental set-up microwave treatment at 98°C for 20 min resulted in a reduction of 4.8 log units of the total number of mesophilic bacteria, while the IR treatment showed a 1 log unit lower reduction for the corresponding temperature and treatment time. Microwave and IR heating created different temperature profiles and moisture distribution within the paprika sample during the heating up part of the process, which is likely to have influenced the decontamination efficiency. The results of this study are used to discuss the difficulties in comparing two thermal technologies on equal conditions due to differences in their heating mechanisms.

## Introduction

Herbs and spices are important food ingredients. However, while they contribute positively to the sensorial properties of the foods, they also often constitute a microbial hazard due to poor sanitation during growth, harvest, drying, and storage. Various bacteria, such as *Salmonella*, *Bacillus cereus*, *Clostridium perfringens*, and *Escherichia coli* are often found on herbs and spices (Banerjee and Sarkar, 2003; Sagoo et al., 2009). The low water activity in dried herbs and spices creates an unfavorable environment for survival of many kinds of vegetative bacteria, which often results in high numbers of bacterial spores such as *Clostridium* and *Bacillus* species (Spruthi, 1980). High amounts of spores in dried spices form a potential microbial hazard since, e.g., *Bacillus* species are known to have high resistance to heat treatments, which may result in germination of surviving spores when spices are added to high water activity foodstuffs (Daelman et al., 2013).

A few non-thermal decontamination technologies have been applied on dried herbs and spices on industrial scale. Unfortunately, these technologies have so far either had disadvantages from a health perspective (ethylene dioxide) or found poor consumer acceptance (γ-irradiation). This has resulted in steam treatment as the most accepted decontamination technology of today in Europe (Schweiggert et al., 2007). However, since steam treatment has important impact on sensorial properties, there is a continuous interest to investigate other methods. Alternative thermal decontamination methods could be microwave heating (Dehne et al., 1990; Dehne and Bögl, 1993; Emam et al., 1995; Legnani et al., 2001; Dababneh, 2012) or infrared (IR) heating (Staack et al., 2008a; Erdoğdu and Ekiz, 2011, 2013; Eliasson et al., 2014). Both IR and microwave heating show attractive properties, above all fast direct heating of the material, which probably could be used in order to reduce processing time, compared to conventional technologies, and thereby potentially also preserve quality characteristics in a better way.

The heating mechanism of IR radiation, corresponding to the wavelengths 0.76 μm–1 mm of the electromagnetic spectrum, is based on the changes of rotations and vibration of molecules, which leads to an energy absorption that further is transferred to heat when the molecules return to their normal state. IR heating is divided into near (0.76–2 μm), medium (2–4 μm), and far (4–1000 μm) IR. The application of different IR wavelength regions affects the amount of transmitted energy, rate of temperature increase and penetration depth. IR heating has a limited penetration depth, about 0.31–4.76 mm dependent on food product and applied wavelengths, and therefore IR is mainly considered a surface heating technology (Skjöldebrand, 2001).

Microwaves are a portion of the electromagnetic spectrum with wavelengths ranging from 1 mm to 1 m and frequencies between 300 and 300 GHz. Microwave energy has been used in food processing applications mainly due to its ability to cause fast volumetric heating that penetrates considerably into the bulk of the material. The heating mechanism of microwaves is based on the interaction between their electric fields and matter, which causes movement of dipoles and ions, a movement that finally translates into heat. The resulting heating depends on many factors. Not only food-related properties, such as moisture content, density, dielectric properties and temperature, are matters of important influence, but also aspects related to the actual design of the microwave heating device. Microwaves penetrate into the heated material—hence they have the potential to cause a more homogenous heating than many other heating methods. Nonetheless, variations of temperature within the food mostly do occur to some extent (Meredith, 1998); these variations are often referred to as hot- and cold-spots and are seldom easily foreseen without thorough investigations.

While a number of studies already investigated IR or microwave decontamination of herbs and spices (Dehne et al., 1990; Dehne and Bögl, 1993; Emam et al., 1995; Legnani et al., 2001; Staack et al., 2008a; Erdoğdu and Ekiz, 2011, 2013; Dababneh, 2012; Eliasson et al., 2014) only a few made comparative studies where one applied technology is compared to another one. IR heating has been compared to the combined effect of IR heating followed by non-thermal ultraviolet treatment (Erdoğdu and Ekiz, 2011). Microwave heating has been compared to non-thermal methods like γ-radiation (Vajdi and Pereira, 1975; Emam et al., 1995; Legnani et al., 2001), but to our knowledge none so far compared the results of two different thermal technologies such as IR and microwave heating for decontamination of spices. Therefore the objective of the present study is to investigate the differences in decontamination efficiency between IR and microwave heating, applied on paprika powder. Since both technologies have been mentioned in the literature as alternative decontamination methods, there is an interest to make a comparison and examine differences in heating mechanism and its influence on temperature profiles, water activity and decontamination. Such studies are important since they can help providing the industry with knowledge for taking decisions concerning appropriate decontamination technologies for spices.

## Materials and Methods

### Preparation of the Raw Material

Paprika powder with a moisture content of 5.6% and water activity of 0.38 was provided by Juan José Albarracin (Murcia, Spain). Prior to the IR and microwave treatments the water activity of the paprika powder was increased to 0.88. This water activity was considered favorable in spices with regards to decontamination efficiency, based on data from previous publications (Staack et al., 2008a; Eliasson et al., 2014). Also pre-trials with dry unconditioned paprika powder showed difficulties in creating a fast and homogenous heating by both IR and microwave heating. The adjustment of the water activity was done in a climate chamber (Vötsch VCL 7010, Balingen-Frommern, Germany) of relative humidity 88% and temperature 20°C. Three trays, of size 21 × 30 cm, containing 150 g of paprika powder each was placed in the climate chamber for 1.5 days. The water activity was controlled by removing samples from the top and bottom of each tray. The samples from the three trays were mixed prior to the IR and microwave treatment.

### Infrared and Microwave Treatment of the Paprika Powder

Infrared respectively microwave heating was used for heating up the paprika sample to 98°C. After the heating up, the sample was moved to a conventional oven with constant temperature, to keep the desired temperature of the paprika sample for holding times of 10 and 20 min. The IR and microwave heating was thus limited to the heating up period. Nonetheless, this constitutes a logical process, provided the sample has been homogenously heated during the heating up period. After that time, only a low power of IR or microwaves would be needed to keep up temperature. In addition, if the sample remains in a cold place, continuous application of microwaves or IR radiation would most likely result in a process that is difficult to control, since cooling happens on the surface of the sample due to the fact that the surrounding air is not heated by IR (Olsson, 2005) or microwaves (Ohlsson, 1999); this results from both methods being radiative in their nature, thus causing direct heating of the food. On the contrary, application of hot-air circulation means adding heat continuously where it is needed during this part of the process.

Effort was made to design the experiments in such a way that IR and microwave treatments would be as comparable as possible. Usage of the same amount of paprika powder (66 g), the same geometry of the heating unit (Petri dish of diameter 11 cm and height 1.5 cm, with cover) and thereby a density of 0.46 g/cm^3^, combined with an identical design of the holding time (10 and 20 min of constant temperature), using an conventional oven (Garomat Electrolux, Stockholm, Sweden), ensured that the samples experienced same conditions during the holding time regardless of the technology used for the heating up. The heating up part of the process was designed in such a way that the paprika sample was heated as fast and homogenously as possible.

The IR heating of paprika powder was performed according to the procedure developed by Staack et al. (2008a) and modified by Eliasson et al. (2014). In brief, 66 g paprika powder was placed in a closed glass Petri dish. The IR transparency of the Petri dish glass cover was measured by the use of a black body. The heat flux received by the black body was measured both without and with the Petri glass cover on the top, and from this the IR transparency of the glass was calculated to be 87%. During IR treatment, the temperature was measured with type-T thermocouples (Pentronic AB, Gunnebo, Sweden) fixed at three different depths of the paprika bed. By the support of a Teflon device, one thermocouple was placed close to the top surface (1.5 mm depth), a second in the middle of the sample (7.5 mm depth) and the third close to the bottom (13.5 mm depth).

**FIGURE 1:**
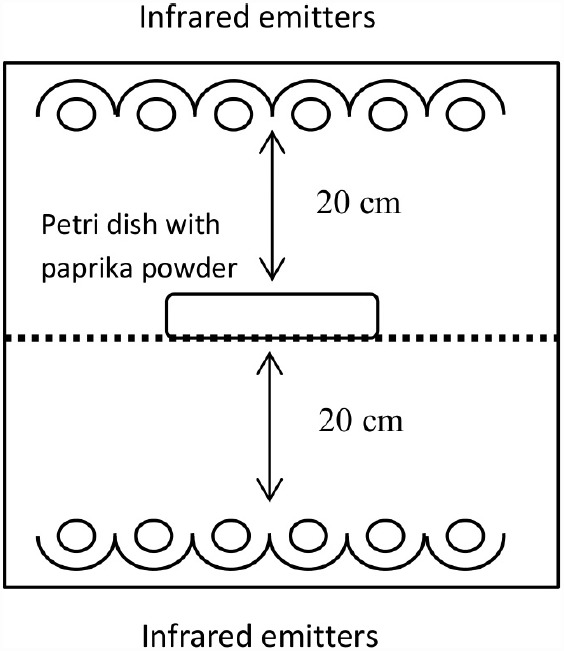
**Schematic picture of the IR heating set-up**.

The IR oven (Ircon Drying Systems AB, Vänersborg, Sweden) was semi-continuous and equipped with six near infrared (NIR) emitters 20 cm above as well as six NIR emitters 20 cm below the sample tray (Figure 1). The sample tray was moving back and forth during the IR treatment. The NIR emitters were of quartz tube type, with tungsten filament and halogen gas, resulting in a maximum IR emission of 1.2 μm (2100°C) at full power level. To reach a homogenous heating up of the paprika sample the IR heat flux was regulated stepwise: 22.6 kW/m^2^ in step 1 for 40s, 11 kW/m^2^ in step 2 for 90 s, and 0 kW/m^2^ in step 3 for 90 s. This recipe was developed by stepwise making small changes in heat flux and time in each experiment. When the desired temperature was reached the settings were duplicated to confirm the result. The indicated heat flux is the value applied from each side. The stepwise regulation was necessary to avoid burning of the surface of the sample and to let the interior part of the sample equilibrate by conduction. The heating unit was moved to the conventional oven during step 3 (0 heat flux), where it then was kept for the desired holding time of 10 or 20 min.

The microwave treatment of paprika powder was performed in the same type of Petri dish as used in the IR treatment. The temperature during the microwave treatments was monitored by four fiber optic probes (Neoptix Inc, QC, Canada) placed at four different radial locations in the Petri dish. The Petri dish with paprika powder was placed in the middle of the microwave oven (Panasonic 1670, Stockholm, Sweden) and a power level of 650 W, 50% of the maximum output, was applied for 60 s. After that the sample was moved to the conventional oven during a step of 0 power, lasting for 30 s. The heating-up experiments with microwaves were repeated five times.

### Visualization of Heat Patterns by Thermal Images

Thermal images of the paprika samples were taken during the IR and microwave heating with a Thermal Tracer TH7700 IR camera (NEC Avio Technologies, Tokyo, Japan). The images were taken at room temperature by removal of samples after 20 and 40 s of heating, as a means to illustrate and evaluate the different nature of the two heating technologies during the heating up part of the process. Thermal images were taken both of the cross-section of the paprika sample and from above.

### Analysis of Water Activity

The water activity of the paprika powder was analyzed in an Aqua-Lab (Decagon Device, Pullman, WA, USA). Different layers of the paprika sample were analyzed after the heating up time (i.e., right after IR or microwave heating), after 10 min holding time and after 20 min holding time in the conventional oven. The Petri dish cover was removed directly after treatment and paprika powder was collected from the top, center and bottom of the paprika bed. Samples were analyzed in duplicates from each layer.

### Microbial Quantification

The naturally occurring microflora in the paprika powder, i.e., aerobic mesophilic bacteria and bacterial spores, was determined before and after IR respectively microwave treatment. As previously mentioned the water activity of the paprika sample was adjusted to 0.88 in a climate chamber. The naturally occurring microflora was also quantified before and after the placement in the climate chamber to ensure the microbial level was not influenced. After each treatment, the Petri dish with 66 g paprika powder was cooled on ice with the cover of the Petri dish kept on. The entire paprika sample was then mixed and 10 g gram sample from this was further transferred and mixed with 90 mL of 0.1% peptone water (0.85% NaCl and 1% peptone; Difco Laboratories/Becton Dickinson, Stockholm, Sweden) and processed in a stomacher for 30 s. The total number of aerobic mesophilic bacteria was determined using viable count on Tryptone Soy Agar (TSA; Oxoid Ltd.) and incubation at 30°C for 72 h (NMKL 2013). Aerobic spores were grown on blood agar base supplemented with 5% horse blood (Sahlgrenska University Hospital, Gothenburg, Sweden) and incubated at 37°C for 48 h. Analysis was performed in duplicates.

## Results and Discussion

### Temperature Profiles

**FIGURE 2:**
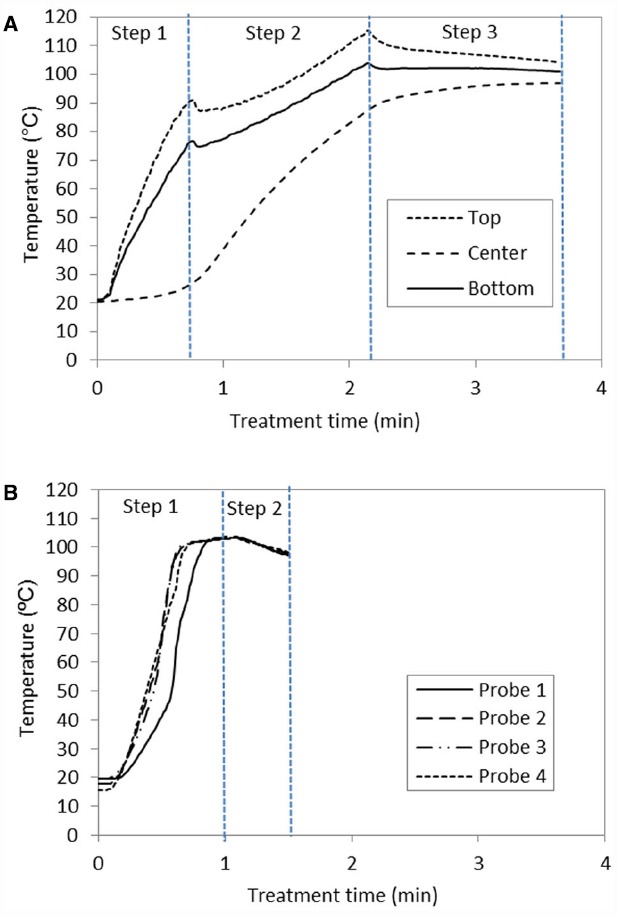
**Temperature profile to reach 98°C in the paprika powder, with an initial water activity of 0.88, treated by (A) infrared heating; step (1) 22.6 kW/m^2^, step (2) 11 kW/m^2^, and step (3) 0 kW/m^2^, respectively (B) microwave heating; step (1) 650 W and step (2) 0 W.** The Petri dish was moved to the conventional oven during step 3 and step 2 for the infrared and microwave treatment respectively.

The IR heat flux was regulated stepwise in order to avoid overheating of the paprika sample’s surface, and thereby reach the treatment temperature as fast and homogenously as possible for the entire sample. The first step of 22.6 kW/m^2^ enabled a fast heating up, while the second step of 11 kW/m^2^ reduced the heating rate at the surface and allowed the interior of the sample to be heated by conduction. After the second step, the difference between the highest and lowest temperature in the sample was 24°C as shown in Figure 2A. Therefore a third step of 0 heat flux was applied to let the temperature equilibrate by conduction, making sure that the temperature in the center of the sample reached the target temperature 98°C. During this step the sample was moved to the conventional oven for holding times of 10 respectively 20 min. The necessary heating up time with IR heating was about 3.7 min to reach minimum 98°C in the entire paprika sample.

The microwave treatment was done by applying a power level of 650 W for 60 s, after which the transfer of the heating unit to the conventional oven was done during the 30 s with 0 power level. The probes reached 98°C after about 45 s; however, taking into account the reduction in temperature when moving the paprika sample to the conventional oven, which can be seen in Figure 2, it was chosen to let the temperature go beyond 98°C during the first step of the heating up with microwaves. To judge from the readings of the four probes, the entire paprika sample appeared to be heated homogenously with the current set-up (Figure 2B). After having applied 650 W for 60 s (in triplicates) the temperature was 103°C with standard variation ± 0.9°C, while, during the transfer time (no applied power) to the conventional oven, the standard variation was 4.8°C around the mean value 98°C.

Both IR and microwave heating are considered as rapid heating technologies. The longer heating up time required for the IR treatment (3.7 min) compared to the microwave treatment (1.5 min), is likely related to the sample thickness 1.5 cm in the present study, combined with the fact that IR heats the surface of the sample, with a penetration depth of a few mm. Erdoğdu et al. (2015) showed experimentally an IR penetration depth of 3.7 mm into paprika powder of a water activity 0.88 when applying near-IR radiation. Such a penetration depth into the paprika powder explains the necessity to reduce the IR heat flux in a stepwise manner to not overheat the surface of the sample and to let the interior part of the sample be heated by conduction, thus affecting the heating up time. In comparison, Staack et al. (2008b) needed a heating up time of about 4.5 min to reach 100°C in paprika powder of water activity of 0.88. In the study by Staack et al. (2008b), the thickness of the sample bed was 1 cm and the IR heat flux was applied from one single side, compared to the two side heating applied in the present study. This means that dependent on the choice of IR set up and sample thickness the heating up time by IR is affected.

After the heating up part of the process, the IR and microwave treated samples were placed inside the conventional oven, keeping 98°C, during the same experimental run. When the door was opened to remove or place samples inside the oven, an air temperature drop of 20–30°C for a few seconds was noted; however, pre-trials had showed that this did not significantly affect the temperature inside the paprika sample itself.

### Thermal Images

**FIGURE 3:**
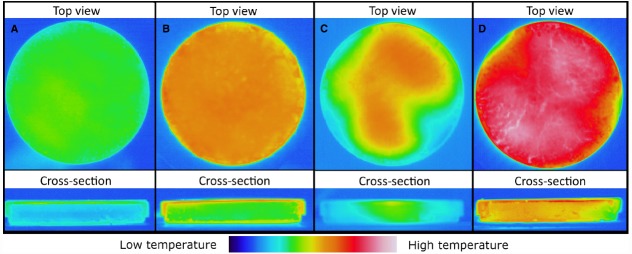
**Thermal images of the paprika powder after IR heating for (A) 20 s, (B) 40 s, and corresponding images of microwave heating after (C) 20 s and (D) 40 s.** Upper row of images are taken from above, and lower row are images taken of the cross-section of the paprika sample. In the first case the petri dish cover was removed after heating; in the latter case the petri dish was split with bottom and cover still in place.

Thermal images were taken during the IR and microwave treatment after interruption of the process and removal of the samples from the IR or microwave oven. This was done to evaluate the heat distribution at two times of the heating-up phase. Based on the total 60 s heating-up time of microwave heating, a time step of 60/3 = 20 s was chosen, resulting in images at times 20 and 40 s, with reference images of IR heating at the same times. The resulting images are shown in Figure 3. The thermal images in the upper row of Figure 3 were taken from above the sample a few seconds after removal of the petri dish cover. The images of the cross-section (the lower row of thermal images) were taken a few seconds after splitting the petri dishes.

As expected Figures 3A,B show that IR generates the highest temperature at the surface of the sample. Furthermore it can be seen that a homogenous heating is achieved over the entire surface (Figure 3B). On the other hand, Figures 3C,D reveal the internal heating nature of microwaves. Seen from above, the microwave treated samples (Figures 3C,D), exhibit a less homogenous surface heating, compared to the IR heating, which can be explained by the formation of hot and cold spots during the microwave heating.

### Reduction of Natural Flora and Water Activity

**FIGURE 4:**
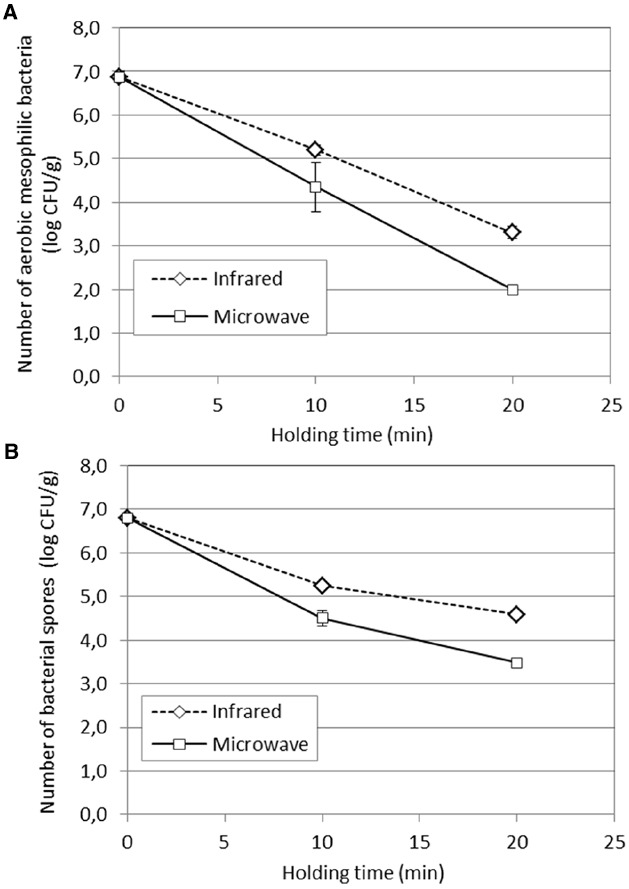
**Reduction of (A) aerobic mesophilic bacteria and (B) bacterial spores in paprika powder, after 10 and 20 min holding times at 98°C.** The paprika powder was conditioned to a water activity of 0.88 prior to the IR and microwave treatments.

The naturally occurring microflora in the paprika batch was 6.80 ± 0.10 log cfu/g for aerobic mesophilic bacteria and 6.87 ± 0.13 log cfu/g for the bacterial spores. The similar level detected on the plates for aerobic mesophilic bacteria and bacterial spores, in combination with visual observations of the colonies, indicated that the natural flora of the paprika batch mainly consisted of bacterial spores. As presented in Figure 4, the best microbial reduction was achieved for the microwave treated paprika powder after a holding time of 20 min, with a 4.8 log units reduction for aerobic mesophilic bacteria and a 3.2 log units reduction for the bacterial spores. The corresponding values for the IR treated samples were 3.8 log units and 2.3 log units. This can be compared to the study by Staack et al. (2008b) where a 4 log units reduction of inoculated *B. cereus* spores was found in paprika powder (water activity 0.88) after 10 min of IR treatment. The less efficient reduction found in the present study could possibly be explained by that a natural flora is more resistant than inoculated *B. cereus* spores, or may possibly be due to differences in the IR heating set up between the two studies.

The differences in the level of reduction between the aerobic mesophilic bacteria and bacterial spores were in the range of 0.13–0.17 log units and 1.37–1.65 log units after 10 respectively 20 min treatment. The natural flora was found to mainly consist of bacterial spores, and thereby the reduction of aerobic mesophilic bacteria and bacterial spores was expected to be similar. The deviation from this expectation for the 20 min treatment, both for IR and microwaves, is likely explained by a better recovery of damaged cells on the blood agar based media used for bacterial spores. This could also explain why the difference between aerobic mesophilic bacteria and bacterial spores are larger after the longer treatment time, when the cells probably are more damaged.

Care was taken to design experiments where the IR and microwave heated samples received a comparable heat-load, by trying to reach 98°C as fast and homogenously as possible. Pre-trials showed that heat treatment at 90°C for 10 min resulted in less than 0.4 log unit reductions of aerobic mesophilic bacteria. Hence it can be assumed that only the last 85 s in Figure 2A, and 42 s in Figure 2B (i.e., when the temperature is above 90°C) result in significant decontamination. The difference between these numbers, 85 and 42 s, in relation to the total holding time is quite small while the resulting decontamination differed by approximately 1 log between the two technologies. This difference is hence likely explained by other factors, such as different variations in distribution of water activity within the samples during treatment and/or differences in the temperature profiles, generated from different heating mechanisms during the heating up part of the process.

As shown by the temperature profiles in Figure 2A and the thermal images in Figures 3A,B, IR is heating the sample from the top and bottom surfaces, while heating of the interior predominantly results from heat-transfer by conduction. The microwave heating seems, in this study, to result in an overall more homogenous profile, compared to the IR heating, due to volumetric heating. However, it should be remembered that the combination of the inherent complexity of microwave heating, including the formation of hot and cold spots, which can be seen in Figures 3C,D, combined with a limited number of measurement points puts a limit to certainty in statements about the overall sample temperature. Another factor that restricted temperature control inside the paprika samples resulted from the fact that, due to technical restrictions, temperature probes had to be removed during movement of the samples from the IR/microwave unit to the conventional oven. Hence temperatures inside the paprika bed could not be recorded during the holding time in the convection oven, even though the air temperature was monitored.

**FIGURE 5:**
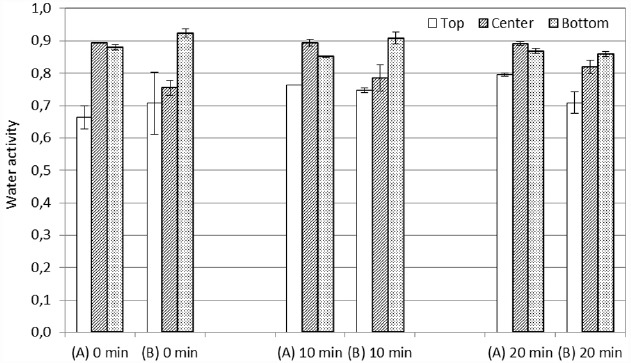
**Water activity of the top, center and bottom layer of the paprika bed after the heating up part of the process (0 min holding time), 10 min holding time and 20 min holding time for the (A) infrared and (B) microwave treated samples.** The overall water activity of the paprika sample was 0.88 before the treatment.

The water activity of the paprika powder was adjusted to 0.88 before IR and microwave treatment. After the heating up part of the process, as well as after 10 and 20 min holding time, the water activity was analyzed in the top, center and bottom layer of the paprika bed. As shown in Figure 5, the heating up with microwaves showed a reduced water activity of 0.75 in the center layer of the sample, while the water activity of the IR treated sample was retained at 0.88 for the corresponding location. The rapid internal heating generated by microwaves creates a pressure-driven flow of liquid water and vapor to the surface of the material (Datta and Ni, 2002; Datta and Rakesh, 2012), thus pushing the water against the wall of the Petri dish. This is a characteristic of the microwave heating, in contrast to IR heating, the latter having more of surface heating properties that do not generate the same pressure-driven flow of moisture to the surface (Datta and Ni, 2002; Datta and Rakesh, 2012). This probably explains both the lower water activity in the center of the sample as well as the increased water activity of 0.92 in the bottom layer, after the heating up with microwaves. However, this pressure-driven flow of moisture is expected to also result in an increased water activity on the top surface of the microwave treated sample. The probable explanation for why this was not confirmed experimentally would be water vapor escaping through the gap between the Petri dish and the cover during treatment. This hypothesis is confirmed by the reduced water activity observed at the top surface also of the IR treated sample.

After holding times of 10 and 20 min, the pattern with a lower water activity in the center of the sample is retained for the microwave treated sample, while the IR treated sample keep the highest water activity in the center of the sample. The differences in the distribution of the water inside the IR respectively microwave treated sample could possibly have influenced the decontamination efficiency. This since previous studies show that the heat resistance of spores is related to the water activity, and its preservation, during heat treatment (Hamanaka et al., 2005; Tiburski et al., 2014).

Another remark can be made on the choice of convection heating during the holding time. As motivated earlier, this is a logical design, although it limits the IR or microwave heating to the heating up part of the process. On the contrary, if one would assume exclusive usage of IR or microwave heating, only small amounts of IR or microwave power would be needed to keep up temperature, unless the samples were intentionally cooled down during the holding time. However, the latter would obviously add a few down-sides to the process, such as poor energy-efficiency.

At a first glance at the results of the present study, one may want to conclude that microwave heating is more effective than IR heating due to the 1 log unit higher reduction of bacteria by microwave heating. However, when comparing the results of two different technologies, one needs to bear in mind that there are technology specific factors that might cause apparent difference in efficiency between two thermal treatments. Applied on the present study, it is likely that such factors are the facts that microwaves generate a volumetric heating while IR heating is better described by surface heating. This creates different heat patterns (Figures 2 and 3) as well as variations in the moisture distribution, and thereby water activity (Figure 5), within the sample. It is likely that these differences have an impact on the resulting decontamination, which thereby could explain the differences in the observed efficiency of the two technologies. Another choice of experimental parameters, for instance a smaller sample thickness or different level of sample compression, could hence possibly have resulted in a more favorable result for the IR heating. In summary, this illustrates the inherent difficulty in designing a representative experimental set-up for comparative studies between two thermal technologies.

From a practical point of view, the difference of 1 log unit between the two heating technologies compared in this study could be set in relation to potential quality gain due to reduced treatment time. To judge from Figures 2A,B, the difference in treatment time would be about 5 min for reaching the same level of decontamination. Further studies are needed to elucidate if this can result in a quality benefit of the paprika powder in terms of sensorial properties.

To our knowledge, IR and microwave heating has not previously been compared in the same study for decontamination of spices. Nonetheless IR and microwave heating have been compared in studies of other applications, such as drying (Chua and Chou, 2005; Sumnu et al., 2005). There are also numerous examples of processes where the technologies have been combined, not the least in baking, with the goal to combine various heating modes for the creation of different temperature and moisture profiles, and thereby tailor the product quality and process efficiency (Datta and Ni, 2002; Datta and Rakesh, 2012).

## Conclusion

As a step in providing knowledge for the industry in their work to look for alternative decontamination methods, the decontamination efficiency of IR and microwave heating, applied on paprika powder has been done. With an adjusted water activity in the product of 0.88, the natural flora of aerobic mesophilic bacteria was reduced by 3.8 log units for IR heating and 4.8 log units for microwave heating, under the conditions of the present experimental set-up. The samples had then been subjected to one heating up phase to reach the desired temperature of 98°C which lasted for 1.5 min (microwaves) respectively 3.7 min (IR), and a subsequent holding time of 20 min at 98°C convection heating.

The observed differences in decontamination efficiency are difficult to explain through a discussion purely based on time and temperature. On the other hand, the present study shows that the two technologies used for the heating-up step cause spatial differences in temperature distribution and consequently in water activity. The theoretically complex relation between total decontamination and spatial variations in water activity and time-temperature makes it difficult to link quantitatively the observed difference in level of total decontamination to the measured values of water activity and temperature. Yet it is not unlikely that the difference in decontamination originates from real variations of water activity and time-temperature. This fact illustrates the difficulties in comparing two thermal technologies on equal conditions due to their inherent differences in heating mechanism.

### Conflict of Interest Statement

The authors declare that the research was conducted in the absence of any commercial or financial relationships that could be construed as a potential conflict of interest.

## Author Contributions

LE performed the IR and microwave treatments and wrote part of the manuscript. SI planned the work, took the thermal images and wrote part of the manuscript. ML designed and interpreted the microbial analysis and reviewed the manuscript. LA helped planning the work and reviewed the manuscript.
